# Validation of the NIOSH Worker Well-Being Questionnaire in Italian Language

**DOI:** 10.1097/JOM.0000000000002835

**Published:** 2023-03-07

**Authors:** Luca Fontana, Pasquale Dolce, Carolina Santocono, Maddalena Annarumma, Ivo Iavicoli

**Affiliations:** From the Department of Public Health, University of Naples Federico II, Naples, Italy.

**Keywords:** worker well-being questionnaire, total worker health, well-being, workplace prevention policies, workers, health surveillance

## Abstract

This study aimed to validate in Italian the Worker Well-Being Questionnaire developed by US National Institute for Occupational Safety and Health.

**Methods:**

The questionnaire was independently translated into Italian by two authors. Translations were compared with obtain a synthesis that was back-translated. Back translations were submitted to an expert committee to produce a final version of the questionnaire. The Italian version, after being pretested, was administered guaranteeing anonymity to a total sample of 206 health care workers.

**Results:**

Findings obtained are satisfactory, indicating excellent fit (CFI and TLI values ranged from 0.96 to 0.99, RMSEA values ranged from 0.03 to 0.07), sound internal consistency of scales (Cronbach’s α values exceeded 0.7) and factor structures consistent with theory.

**Conclusions:**

Italian version of the questionnaire is faithful to the original and allows for efficient and robust measurement of workers’ well-being.

## Abstract 2

The TWH® program is an extremely promising model for developing innovative policies/strategies to ensure that OSH evolves to keep pace with the changing world of work. In this regard, our findings provide an important contribution to full applicability of WellBQ by confirming its reliability and effectiveness in measuring workers’ well-being.

**LEARNING OUTCOMES**

- Identify the fundamental principles that characterize the *Total Worker Health*^®^ model;
- Evaluate the use and acquire skills in the administration of the Worker Well-Being Questionnaire (especially in Italian working populations).

In the constitution of World Health Organization (WHO), adopted by the International Health Conference held in New York in 1946, health was defined as “a state of complete physical, mental and social well-being and not merely the absence of disease or infirmity.”^1^ In this regard, 40 years later, on the occasion of the first International Conference on Health Promotion held in Ottawa in 1986, the WHO reported in the Ottawa Charter for Health Promotion that “To reach a state of complete physical, mental and social well-being, an individual or group must be able to identify and to realize aspirations, to satisfy needs, and to change or cope with the environment. Health is, therefore, seen as a resource for everyday life, not the objective of living.”^2^ Therefore, it is quite intuitive that to be considered healthy, people should be able to fulfill their aspirations and meet their needs in every field of their existence, including communities, home, as well as working life. Indeed, relatively to the last point, if we keep in mind that usually a work shift lasts about 8 hours, it follows that people spend a third of their daily day in the workplace. Consequently, according to what is defined by the WHO, it is unthinkable that a worker can be considered healthy if he is not able to fulfill himself and adequately deal with the work environment, because these prerogatives define the possibility of achieving a state of complete physical, mental and social well-being.^1^

Traditionally, the principle of protecting workers’ safety and health has translated into practice in the implementation of an occupational risk assessment and management system whose main purpose has always been to ensure safe working conditions and, at the same time, protect workers from adverse health effects that may result from occupational exposures.^3,4^ However, despite the efforts made over the years, recently the International Labour Office estimated that globally accidents and occupational diseases still cause more than two million deaths annually.^5^ Therefore, even though the aforementioned approach still represents the foundations of the Occupational Safety and Health (OSH) management system, recently in the scientific community a new awareness has made its way relatively of having to expand the classic OSH system by progressively moving toward a holistic model which, by exploiting an integrated approach and multidisciplinary intervention strategies, can concretize the health concept as defined by the WHO.^6–8^

Indeed, it is widely recognized and debated that the intrinsic characteristics of work, the workforce, and the workplace are constantly evolving under the influence and thrust of multiple factors such as technological advances, globalization outcomes, shifts in demographics (i.e., aging and gender issues), migration, climate changes, informal work and, not least, the pandemic.^9^ On the whole, these elements can have serious consequences on the overall quality of work, the well-being of the workforce, and the integrity of workplaces by modifying and negatively affecting various parameters such as work organization demands, paid sick leaves, wages, psychosocial hazards, and hours of work.^6,10–12^ Hence, in this context, these work-related factors, being able to influence workers’ health and the well-being of their families and social context, should be at the heart of the OSH system concerns which, in this perspective, should be able to go beyond the analysis of traditional occupational risk factors alone (e.g., exposures to chemical, physical, or biological agents).

Furthermore, in recent years, more and more scientific evidence has been accumulating to support the fact that some of these work-related factors (e.g., shiftwork, long working hour, sedentary work, workloads) can contribute to the onset or worsening of several disorders, pathologies, and syndromes, such as obesity, diabetes, cardiovascular diseases, cognitive, and sleep disorders.^13–21^ Consequently, there is a clear need to identify an evaluation model that takes into account both occupational and/or work-related risk factors and personal health conditions and/or lifestyle factors and that is able to analyze the possible interactions and reciprocal influences between all these elements. In this regard, the *Total Worker Health*^®^ program proposed by the US National Institute for Occupational Safety and Health (NIOSH) is consistent with this perspective since its main aim is to protect workers on and off the job by defining policies, programs, and practices that not only protect them from accidents at work and occupational diseases but at the same time can implement effective health promotion programs that improve their overall well-being.^22,23^ For example, recently in Italy the Total Worker Health (TWH) approach has received a great deal of attention, not only from prevention professionals, but also and especially from legislators. In fact, this model is cited, strongly enhanced and largely endorsed by the Italian Ministry of Health within the National Prevention Plan (PNP) for the 5-year period 2020 to 2025.^24^ In detail, in this document, it is recognized that TWH model is an effective intervention methodology to reduce work-related injuries and occupational diseases but, at the same time, to make the workplace a health-promoting environment.^25^

Obviously, to identify the most effective intervention policies and action strategies capable of realizing the theoretical and fundamental principles of the TWH approach, innovative and adequate tools are needed to collect a large number of reliable and high-quality information through which characterize the main dimensions of workers’ well-being and thus reach a widely agreed definition of this concept. In this context, the NIOSH recently released the Worker Well-Being Questionnaire (WellBQ) that is a self-report survey instrument to broadly assess worker well-being and that can find several applications in research, policy, and practice.^3,26^ Therefore, this study aimed to validate, in a sample of health care workers, the Italian version of the WellBQ questionnaire to allow it to be widely used also in our country to collect data useful for defining evidence-based policies and strategies that can contribute to improving the well-being of workers. In fact, the validation of this tool in the Italian language is an urgent matter, especially in the light of what is referred to in the PNP 2020–2025, which is likely to lead to a wide application of the TWH principles and, of course, consequently also of its main instruments, such as the WellBQ questionnaire.

## MATERIALS AND METHODS

### Description of the NIOSH Worker Well-Being Questionnaire

The WellBQ has recently been developed and validated by NIOSH researchers and at present, in the context of the TWH model, it represents the most comprehensive survey instrument for assessing and measuring worker well-being.^3,26^ In this regard, to obtain reliable and high-quality information that can then be useful in informing and guiding the various stakeholders in devising proactive policies and applying effective intervention strategies, it is first and foremost crucial to reach a consensus on what worker well-being actually is. The NIOSH has been focusing on this issue for several years and following a process of developing a conceptual framework for workers’ well-being has defined it as an “integrative concept that characterizes quality of life with respect to an individual’s health and work-related environmental, organizational, and psychosocial factors. Well-being is the experience of positive perceptions and the presence of constructive conditions at work and beyond that enables workers to thrive and achieve their full potential.”^3^ Consequently, based on this framework and the aforementioned definition, NIOSH designed a new survey instrument (that is the WellBQ) to comprehensively measure worker well-being and that may be used widely in today’s different workplaces.^26,27^

**FIGURE 1:**
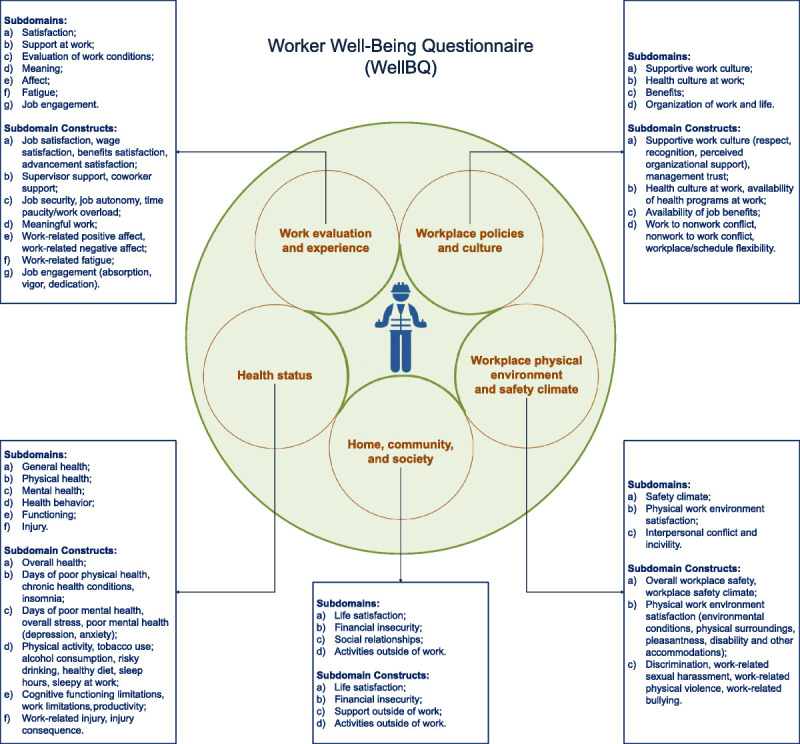
Domains, subdomains, and subdomain constructs of the WellBQ. The work evaluation and experience domain includes 4 scales and 10 single items; The workplace policies and culture domain includes 3 scales, 2 indices, and 3 single items; The workplace physical environment and safety climate domain includes 4 scales and 3 single items; The health status domain includes 4 scales, 2 indices, and 13 single items; The home, community, and society domain includes 1 scale, 1 index, and 2 single items (modified from Chari et al., 2022).^26^

This innovative tool consists of 126 total items distributed in 24 subdomains and 52 subdomain constructs, thus providing 5 indices and 16 scales (Fig. 1).^27^ Clearly, at this stage of development, the WellBQ has some limitations, for example, the scarcity of data currently available from the administration of the questionnaire does not allow to develop algorithms for the definition of summary scores to typify workers’ well-being. In addition, it is important to underline that the results provided by this tool do not in any way allow absolute or clinical judgments to be drawn on the well-being of workers, nor, moreover, does the questionnaire identify thresholds or cut-off scores that can signal the need to intervene with actions aimed at improving the well-being of workers.^27^ Nonetheless, the use of WellBQ in the workplaces can be particularly useful for performing a baseline assessment and thus defining internal benchmarks for a company or a specific homogeneous group of workers (within that company), for assessing changes in well-being following the application of specific interventions, but also for identifying, by comparing results between worker groups within the same facility, critical areas where action can be taken.^27^

### Translation of the Worker Well-Being Questionnaire

**FIGURE 2:**
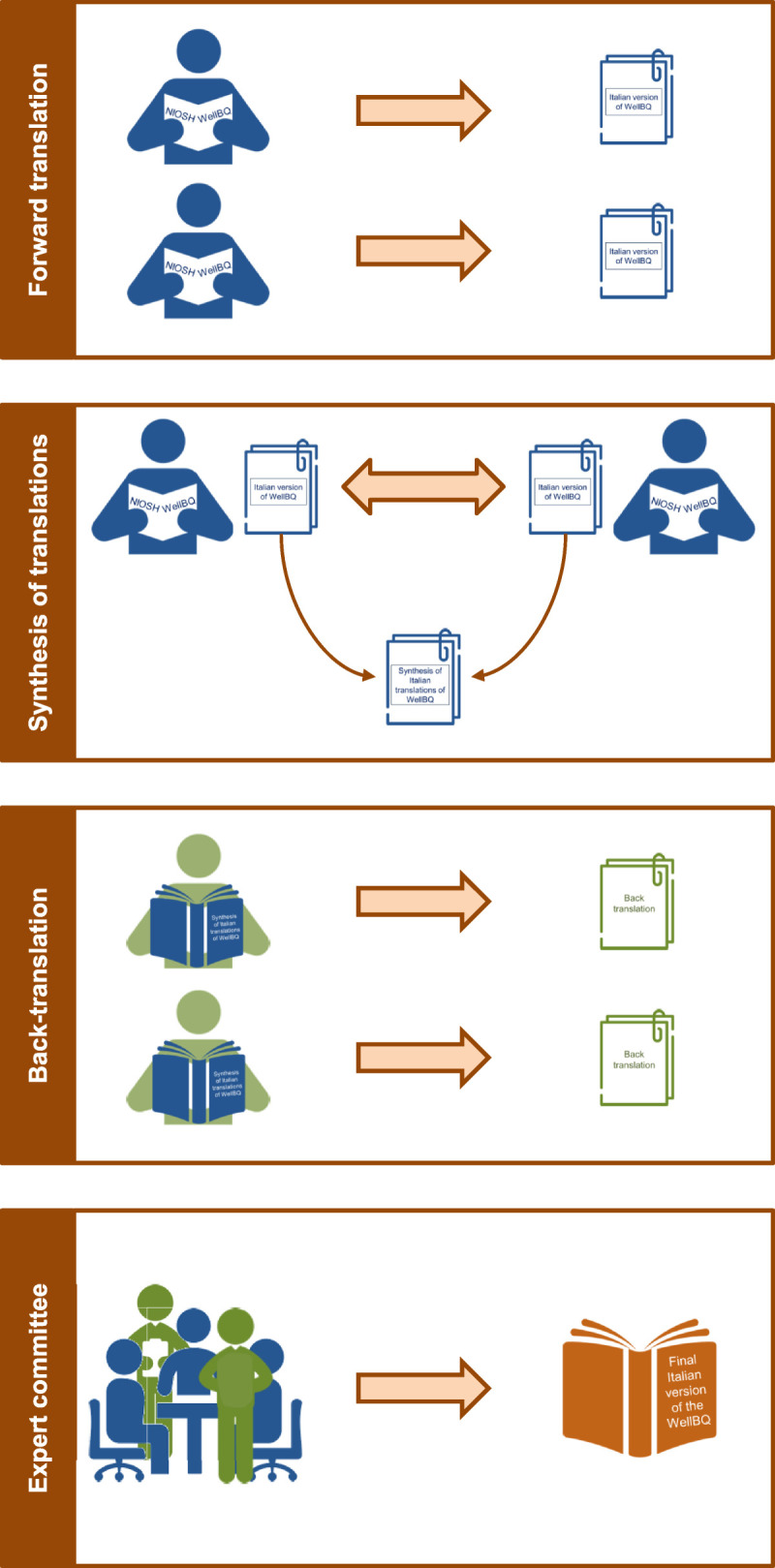
Methodology and main steps carried out to translate the WellBQ.

The translation and the following validation of the WellBQ were conducted, through different phases, following the main indications provided by Beaton et al.^28^ In this regard, the first step (forward translation) consisted of the translation in the Italian language of the complete questionnaire (including instructions, items and answer options). In more detail, the forward translation was initially carried out by two authors (with excellent language skills in both English and Italian) who translated the questionnaire independently of each other, thus producing two distinct translations (Fig. 2).

Subsequently, the two translations were compared with each other and the authors, after identifying the phrases or expressions from the WellBQ that had not been translated in the same way, proceeded to check together which translation was more faithful to the original version and then produced a final synthesis of the Italian translation. The adequacy and validity of this synthesis, especially in terms of content equivalence and preservation of the original meaning of the questionnaire items, were verified through a back-translation from Italian to English. Therefore, two English fluent language subjects, totally blind to the original version of the tool, performed a translation of the Italian synthesis of the questionnaire that was obtained in the previous phase (back-translation). This validity checking process is very important since it allows to identify inconsistencies and/or conceptual errors, pointing out unclear wording and ensuring that the translated version is coherent in content with the original version.^26^ Finally, the two back-translations (together with all the other translations made in the previous phases) were submitted to an expert committee to obtain a final version of the questionnaire for field testing. In this context, the expert committee consisting of authors of this article and the translators involved in the earlier stages, examined all the documents and reached a final consensus on any discrepancies found between the English version of the WellBQ and the Italian translated version. In particular, the expert committee evaluated their semantic, idiomatic, experiential and conceptual equivalence. For each translation proposal, it was examined whether the target concept was explored and expressed correctly, whether discrepancies were identified, whether the translators’ choices were taken into account and whether a consensus was reached. At this stage of the linguistic validation process, each expert contributed his or her own point of view and background and, through fruitful discussion and brainstorming, ensured that the tool was appropriate for its purpose and context.

### Pretesting, Participants, and Data Collection

Once the final Italian version of the WellBQ was defined using the abovementioned methodology, it was field-tested in 32 subjects from the target setting (e.g., physicians, nurses and technical personnel) who belonged to the Occupational Medicine section of our university hospital. Therefore, each of the participants in the pretesting phase, after completing the questionnaire, was interviewed to check for any difficulties in understanding the meaning of one or more questions and/or answers or in filling out the tool. In this regard, it is important to emphasize that this step is essential to understand how, in an applied situation, the subject to whom the questionnaire is administered interprets and reacts to the WellBQ questions and thus the pretesting provides a certain measure of quality in content validity. Moreover, to assess the comprehensibility, applicability, and compliance of the health care professionals, we also evaluated the distribution of the answers to identify any high percentages of missing items (or single answers) that could therefore be an indication of a criticality in the clarity or compilation of the WellBQ Italian version, thus suggesting the need to explore in more detail or more simply some terms or concepts.

This validation study was conducted on 206 health care workers (e.g., physicians, nurses, and technical personnel such as biomedical laboratory, radiology and perfusion technicians) employed in an Italian university hospital located in a metropolitan city in Southern Italy. All the workers enrolled in the study underwent the mandatory periodic medical examination scheduled in the context of medical health surveillance according to the Legislative Decree n. 81/08. During the health surveillance medical examination sociodemographic data, occupational characteristics, anamnestic data and information about recreational habits/lifestyle and diet were collected. In addition, between September and December 2022, study participants were asked to self-administer the Italian version of the NIOSH WellBQ and the collection of completed questionnaires was carried out guaranteeing anonymity. In this regard, it is worth emphasizing that, before the administration of the questionnaire, adequately trained and instructed medical personnel provided the subjects who agreed to participate in the study with explanations regarding the correct fulfillment of the WellBQ, its meaning and the items it contained. Therefore, after being informed about the aims of the study and the related data processing procedures, subjects declared their written informed consent to both participation in the study and possible future publication of the data in an anonymous and collective form. We excluded workers who refused to sign the informed consent. The protocol study was approved by the local Ethics Committee of the University Federico II–AORN Cardarelli, Naples, Italy (protocol 16082/2022).

### Statistical Analysis

To evaluate the construct validity of the Italian version of WellBQ, three order confirmatory factor analysis was performed for each domain. We evaluate the factor model fit through the following indices: root mean square error of approximation (RMSEA), comparative fit index (CFI), and Tucker–Lewis index (TLI). Values of RMSEA <0.08 and TLI and CFI >0.95 are indicative of a good fit.^29^ The internal consistency for scales with two or more items was evaluated through the Cronbach’s alpha (values >0.7 are indicative of good internal consistency). Then, relationships among domain, subdomain and constructed were evaluated estimating the standardized loadings (λ). By default, the first loading of each equation was set to 1 and the corresponding standardized loading was reported.

The diagonally weighted least squares was used for estimation and the weighted least squares to compute robust standard errors for statistical tests. Concurrent, convergent, and discriminant validity of questionnaire measures were assessed computing Spearman’s correlation coefficient among items and scales. Significance level was set to 0.05 in all tests. All analyses were performed using the R statistical software and, in particular, the Lavaan R package was used for Factor Analysis.^30^

## RESULTS

### Sample Characteristics

Considering the employed statistical methodology, which typically require a sample size of at least 200 participants,^29^ in the present study a sample of 206 subjects was analyzed. Participants were almost equally distributed by sex (48.5% females and 51.5% males). Respondents in the age range of 25–44 years were the most represented (37.9%), while 65 (31.5%) were in the age range of 45 to 55 years and 63 (30.6%) were older than 55 years. Nearly 35% of the respondents were physicians, 67 (31%) were nurses and 67 (32.5%) were technicians. Most of the participants (98.54%) completed high school (83.98% possessed a bachelor’s or higher degree), worked full-time (99.03%) and had a working length of more than 20 years (47.57%). In 2020, according to data provided by the Italian Ministry of Health, in Italy 617.466 workers were employed in the National Health System. In greater detail, 103.092 (16.7%) were physicians (49.8% females and 50.2% males), 264.686 (42.8%) were nurses (77.8% females and 22.2% males) and 109.814 (17.8%) were technical personnel (64.6% females and 35.4% males).^31^ Furthermore, in 2018, the Italian National Institute of Statistics showed that National Health System workers had a mean age of 50.7 years (52.3 years for males and 49.9 years for females), 93.9% had a permanent contract and 91.5% worked full-time.^31,32^

### Domain 1: Work Evaluation and Experience

The Italian version of the work evaluation and experience domain showed an excellent model fit providing values of RMSEA, TLI and CFI of 0.06, 0.96, and 0.97, respectively. Internal consistency for the 2 (or more)—item scale was good for the work-related positive and negative affect scales (Cronbach’s α was 0.88 and 0.84, respectively), slightly low for meaningful work (α = 0.66) but not very satisfactory for job engagement (α = 0.53). In this regard, it is plausible to assume for both scales that the moderate correlation between the individual items is due to the peculiarities of the health care professional field in which the vocational aspect toward one’s work is definitely high and may therefore influence the frequency of response in some items such as in questions Q10 “The work I do is meaningful to me,” Q11 “The work I do serves a greater purpose” or Q14 “My work inspires me.”

**FIGURE 3:**
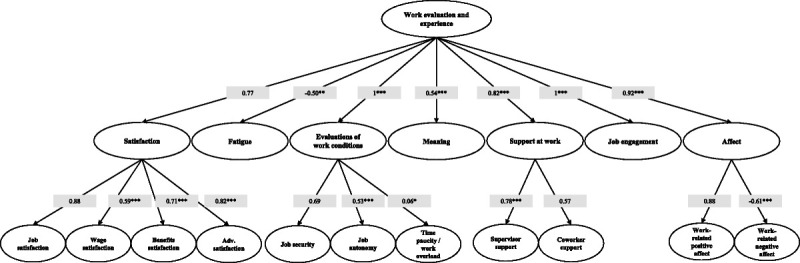
Path diagram and loadings estimates of the third-order factor model for the Italian version of “Work evaluation and experience” domain. CFI = 0.97, TLI = 0.96, RMSEA = 0.06; Cronbach’s α: meaningful work = 0.66, work-related positive affect = 0.88; work-related negative affect = 0.84; Job engagement = 0.53; *P < 0.05, **P < 0.01, ***P < 0.001.

All the different subdomains contained in this part of the questionnaire were found to be positively and significantly related to the reference domain (all second-order factor loadings were positive and statistically significant—*P* < 0.001—see Fig. 3), except for the subdomain of “work-related fatigue”, which had a significant and inverse relation with the domain (λ = −0.5, *P* < 0.01). This finding seems consistent with the construction of the questionnaire since as worker’s overall perception of “work evaluation and experience” domain would increase, the work-related fatigue decreases. The strongest relations were observed for the “evaluation of work conditions”, “affect” and “job engagement” subdomains, thus suggesting that these areas play a decisive role in the domain construction. With regards to the relationships between subdomains and related constructs, all subdomains were positively related to the underlying constructs, except for “work-related negative affect” construct that presented an inverse relation with the related domain (consistently with the subdomain construction and its meaning). All relations were statistically significant, except one (see Fig. 3).

### Domain 2: Workplace Policies and Culture

**FIGURE 4:**
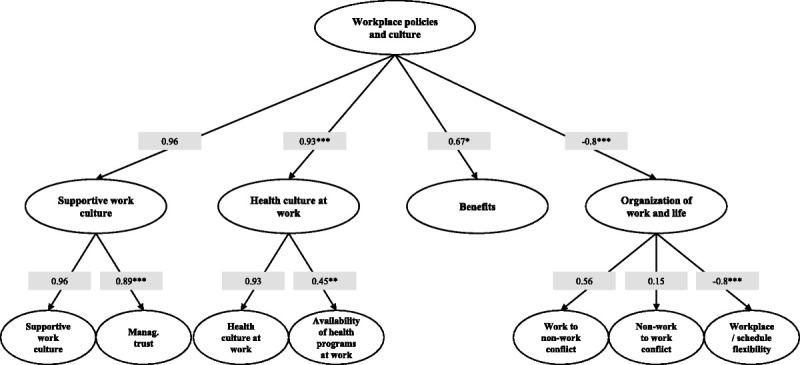
Path diagram and loadings estimates of the third-order factor model for the Italian version of “workplace policies and culture” domain. CFI = 0.98, TLI = 0.98, RMSEA = 0.06; Cronbach’s α: supportive work culture = 0.92, health culture at work = 0.76; workplace/schedule flexibility = 0.69; *P < 0.05, **P < 0.01, ***P < 0.001.

With regard to the validation of the second domain of the Italian version of the WellBQ, we found a RMSEA of 0.06, a TLI of 0.98 and a CFI of 0.98 (Fig. 4). All the scales showed a good internal consistency, obtaining a Cronbach’s α values of 0.92 and 0.76 for the “supportive work culture” and the “health culture at work” scales, respectively, whereas the two-item scale of “workplace/schedule flexibility” had a Cronbach’s α value of 0.69, very close to the commonly used threshold. Three of the four subdomains in this section of the questionnaire were positively and significantly related with the corresponding domain (λ = 0.96 for supportive work culture, λ = 0.93, *P* < 0.001, for “health culture at work” and λ = 0.67, *P* < 0.05 for “benefits”). The “organization of work and life” subdomain exhibited a negative and statistically significant relation with the overlying domain. Two of the three constructs that contribute to this subdomain (“work to nonwork conflict” and “nonwork to work conflict”) were positively related to it, while “workplace/schedule flexibility” showed a negative relation with the subdomain (λ = −0.8, *P* < 0.001). However, the relation between the subdomain and the construct “nonwork to work conflict” was not statistically significant. Overall, these results were consistent with the theories and considerations of occupational medicine since the finding of a high level of conflict between the work environment and personal life significantly raises the score of this subdomain, whereas a high score at the workplace/schedule flexibility scale lowers it. All other subdomains were positively and significantly related to the corresponding constructs (see Fig. 4).

### Domain 3: Workplace Physical Environment and Safety Climate

**FIGURE 5:**
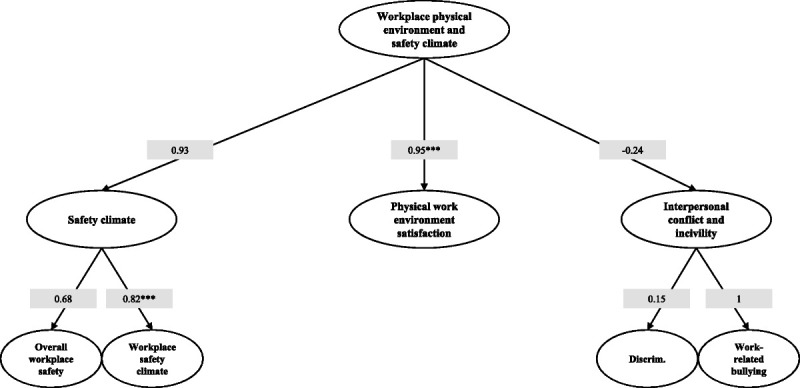
Path diagram and loadings estimates of the third-order factor model for the Italian version of “Workplace physical environment and safety climate” domain. CFI = 0.98, TLI = 0.97, RMSEA = 0.07; Cronbach’s α: workplace safety climate = 0.95, physical work environment satisfaction = 0.87; discrimination = 0.71, work-related bullying = 0.7; *P < 0.05, ** *P* < 0.01, *** *P* < 0.001.

Similar to the previous ones, the validation of third domain (Fig. 5) also provided excellent results showing a very good model fit (RMSEA = 0.07; TLI = 0.97; CFI = 0.98). In this domain, all the scales showed a good internal consistency since Cronbach’s α values were 0.95, 0.87, 0.71, and 0.7 for workplace safety climate, physical work environment satisfaction, discrimination and work-related bullying scales, respectively. Concerning the relationship between the domain and the underlying subdomains, we found a statistically significant correlation with the “physical work environment satisfaction” domain (λ = 0.95, *P* < 0.001), but the “interpersonal conflict and incivility” subdomain presented a nonsignificant negative relation (λ = −0.24) with the reference domain (as the score of this subdomain increases, the overall value of the “workplace physical environment and safety climate” domain decreases). Although this relation was not statistically significant, it is entirely consistent with the principles, theories, and practical observations of the occupational medicine discipline. In addition, regarding this subdomain, it is important to point out that in the validation model, we excluded the “work-related sexual harassment” construct, since all participants provided a negative answer to item Q37 and thus there was no variability in the responses and, similarly, we excluded Q38 item because of only one affirmative response. Finally, all the subdomains were positively related with the relative constructs (Fig. 5).

### Domain 4: Health Status

**FIGURE 6:**
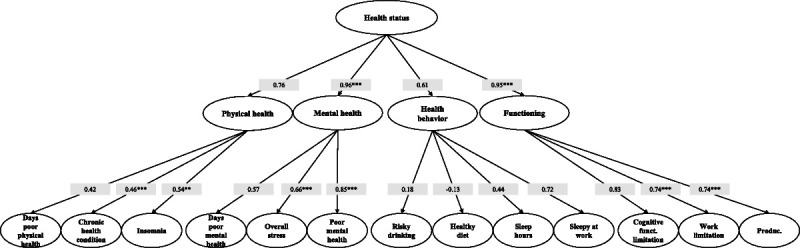
Path diagram and loadings estimates of the third-order factor model for the Italian version of “health status” domain. CFI = 0.99, TLI = 0.99, RMSEA = 0.03; Cronbach’s α: overall stress = 0.83, poor mental health = 0.86, physical activity = 0.53, productivity = 0.75, *P < 0.05, **P < 0.01, ***P < 0.001.

Statistical analyses conducted on domain 4 showed a very good model fit (Fig. 6) with RMSEA = 0.03, TLI = 0.99 and CFI = 0.99. However, it should be considered that in our validation model we have excluded some subdomains (“general health” and “injury”) and constructs (“physical activity”, “tobacco use”, “alcohol consumption”, “work-related injury”, and “injury consequences”). In detail, the “injury” subdomain and its constructs were removed from the validation model due to the extremely low variability in the answers provided by the participants. In this regard, only 5.8% reported having had a work-related injury in the last 12 months, and of these, less than 50% stated that they had suffered consequences as a result of the accident they had encountered. For the same reason, we also excluded the “alcohol consumption” construct since, after coding the participants’ answers according to the instructions provided by NIOSH (code more than 14 drinks as 1 and code 14 or fewer as 0 for males and code more than 7 drinks as 1 and code 7 or fewer as 0 for females), all answers were coded as 0. Similarly, we decided not to take the “tobacco use” five-item index into account because of the low frequency of affirmative responses for some modality. Moreover, this variable cannot be included in the model as an index, because a high value of the index does not necessarily correspond to a high use of tobacco With regard to the “physical activity” construct, statistical analysis suggested that in our study the two items of the scale were not strongly correlated with each other and, also in this case, it is likely to assume that this result is due to the inherent characteristics of the work activity performed by the population enrolled in the study. In fact, most of the participants (especially technicians and nurses) perform work that requires significant physical exertion, and this element may have influenced the frequency of responses in the two items on this scale. With regard to the subdomain of “general health”, its inclusion in the validation model strongly decreased the values of CFI and TLI and for this reason it was excluded. In this regard, it is plausible to assume that the low coherence of this single item is attributable to the generality of the question itself.

As can be seen from Fig. 6, all scales, with the exception of the “physical activity” scale (α = 0.53), showed a good internal consistency and all the subdomains were positively correlated to the “health status” domain (although λ for Health Behavior was not statistically significant). In this regard, it is noteworthy to point out that, among the various subdomains, those that would play a greater role in the construction of the overlying domain are “mental health” and “functioning” (λ = 0.96 and λ = 0.95, *P* < 0.001), which would then represent the priority areas of intervention to seek better “health status” scores. Similarly, taking into consideration the relationship between subdomains and constructs, the results of the analyses showed that, except for the health behavior subdomain, all constructs are positively and significantly related to the corresponding subdomain (“chronic health conditions” [λ = 0.46, *P* < 0.001] and “insomnia” [λ = 0.54, *P* < 0.01] for “physical health”, “overall stress” [λ = 0.66, *P* < 0.001] and “poor mental health” [λ = 0.85, *P* < 0.001] for “mental health” and “work limitations” [λ = 0.74, *P* < 0.001], and “productivity” [λ = 0.74, *P* < 0.001] for “functioning”).

### Domain 5: Home, Community, and Society

Because this domain is composed of only two single item, one 2-item scale and one 7-item index, a factor analysis did not make sense. Following the original paper,^26^ only Cronbach’s α was computed for the two-item scale and it was equal to 0.62, showing that the internal consistency of the scale was slightly low, but it should be considered that this is a scale of only two items.

### Correlations Among Selected Scales and Items

Similarly to the study by Chari et al,^26^ we analyzed the correlations among selected scales and items to verify the concurrent, convergent, and discriminant validity of the Italian version of WellBQ. The results of this statistical analysis are shown in Table 1 and are largely superimposable on those obtained in the aforementioned article, demonstrating the existence of associations that are in agreement with the occupational medicine theories and consistent with the occupational health observations and considerations provided by field studies already published in the literature.^26^ The main differences found when comparing our results with those provided by Chari et al,^26^ were observed for the following items and scales “time paucity/work overload”, “meaningful work”, “availability of health programs at work”, “availability of job benefits”, “physical activity”, “tobacco use”, “risky drinking”, “healthy diet”, “support outside of work”, and “activities outside of work”. In this regard, it is plausible to hypothesize that the slight discrepancies in domains 4 (health status) and 5 (home, community, and society) are essentially attributable to social and cultural differences between the populations enrolled in the two studies. Otherwise, in our opinion, it is conceivable that the discordances found in domains 1 (work evaluation and experience) and 2 (workplace policies and culture) are mainly attributable to the peculiarities of the health care professional field and work environment, as mentioned above. The meaning of the most important and significant associations that support the validity of the questionnaire are discussed in the following section.

**TABLE 1:** Correlations Among Select Scales and Items in the Italian Version of the WellBQ

Selected Scales or Items	Job Satisfaction	Life Satisfaction	Work-Related Positive Affect	Work-Related Negative Affect	Job Stress	Overall Stress	Poor Mental Health	Days of Poor Mental Health	Days of Poor Physical Health	Overall Health	Overall Workplace Safety	Low Productivity
Domain 1: work evaluation and experience												
Job satisfaction	1	0.36***	0.58***	−0.46	−0.48***	−0.49***	−0.41***	−0.30***	−0.23***	0.31***	0.41***	−0.32***
Wage satisfaction	0.51***	0.25***	0.27***	−0.21**	−0.36***	−0.35***	−0.24***	−0.12	−0.19**	0.2**	0.32***	−0.14
Benefits satisfaction	0.49***	0.23**	0.37***	−0.37***	−0.41***	−0.38***	−0.31***	−0.3***	−0.21**	0.28***	0.44***	−0.19*
Advancement satisfaction	0.56***	0.3***	0.47***	−0.32***	−0.40***	−0.34***	−0.4***	−0.28***	−0.23***	0.32***	0.47***	−0.15*
Supervisor support	0.47***	0.23**	0.45***	−0.25*	−0.38***	−0.35***	−0.17*	−0.11	−0.22**	0.2**	0.44***	−0.17*
Coworker support	0.37***	0.31***	0.44***	−0.29***	−0.34***	−0.37***	−0.27***	−0.28***	−0.2*	0.34***	0.34***	−0.2**
Job security	0.46***	0.37***	0.42***	−0.4***	−0.50***	−0.47***	−0.32***	−0.3***	−0.26***	0.28***	0.62***	−0.18**
Job autonomy	0.43***	0.2**	0.45***	−0.3***	−0.29***	−0.21**	−0.23***	−0.15*	−0.16*	0.16*	0.21**	−0.17*
Time paucity/work overload	0.07	0.05	0.1	0.17*	0.03	0.0	0.05	0.07	0.07	0.01	0.02	0.15*
Meaningful work	0.32***	0.24***	0.41***	−0.09	−0.02	−0.07	−0.16*	0.0	−0.06	0.16	0.16	−0.18**
Work-related positive affect	0.58***	0.33***	1	−0.49***	−0.45***	−0.4***	−0.55***	−0.35***	−0.34***	0.37***	0.44***	−0.39***
Work-related negative affect	−0.46***	−0.24***	−0.49***	1	0.55***	0.52***	0.44***	0.42***	0.27***	−0.27***	−0.52***	0.4***
Work-related fatigue	−0.35***	−0.33***	−0.35***	0.47***	0.46***	0.45***	0.29***	0.36***	0.3***	−0.29***	−0.37***	0.21**
Job engagement	0.46***	0.18**	0.62***	−0.17*	−0.17*	−0.2**	−0.26***	−0.14*	−0.18*	0.18**	0.12	−0.31***
Domain 2: workplace policies and culture												
Supportive work culture	0.59***	0.25***	0.52***	−0.42***	−0.48***	−0.38***	−0.33***	−0.33***	−0.29***	0.25***	0.53***	−0.22**
Management trust	0.52***	0.36***	0.47***	−0.44***	−0.5***	−0.41***	−0.39***	−0.38***	−0.34***	0.28***	0.56***	−0.28***
Health culture at work	0.44***	0.25***	0.41***	−0.32***	−0.31***	0.25***	−0.26***	−0.32***	−0.26***	0.2**	0.5***	−0.27***
Availability of job benefits	0.12	0.13	0.17*	−0.03	−0.12	−0.06	−0.03	−0.12	−0.16*	0.05	0.07	−0.23***
Availability of health programs at work	0.17	−0.03	0.18	−0.15*	−0.14	0.04	−0.13	−0.14	−0.21*	0.06	0.06	−0.01
Work to nonwork conflict	−0.29***	−0.18**	−0.24***	0.4***	0.58***	0.45***	0.24***	0.25***	0.11	−0.15*	−0.38***	0.18*
Nonwork to work conflict	−0.19**	−0.23***	−0.12	0.11	0.17*	0.37***	0.01	0.01	0.12	−0.22**	−0.18**	0.21**
Workplace/schedule flexibility	0.4***	0.24**	0.41***	−0.4***	−0.46***	−0.45***	−0.25***	−0.16*	−0.17*	0.17*	0.52***	−0.22**
Domain 3: Workplace physical environment and safety climate												
Overall workplace safety	0.41***	0.37***	0.44***	−0.52***	−0.48***	−0.48***	−0.33***	−0.29***	−0.2**	0.34***	1	−0.3***
Workplace safety climate	0.37***	0.3***	0.31***	−0.35***	−0.41***	−0.35***	−0.3***	−0.27***	−0.2*	0.16	0.5***	−0.17*
Physical work environment satisfaction	0.34***	0.27***	0.32***	−0.36***	−0.43***	−0.38***	−0.33***	−0.35***	−0.23**	0.27***	0.53***	−0.29***
Discrimination	−0.24***	−0.17*	−0.28***	0.34***	0.29***	0.3***	0.28***	0.22**	0.24***	−0.21**	−0.22**	0.31***
Work-related physical violence	−0.13	−0.01	−0.12	0.12	0.12	0.12	0.12	0.12	0.13	−0.13	−0.05	0.09
Work-related bullying	−0.27***	0.0	−0.3***	0.3***	0.23***	0.12	0.14*	0.14*	0.16*	−0.09	−0.11	0.06
Domain 4: health status												
Overall health	0.31***	0.37***	0.37***	−0.27***	−0.26***	−0.41***	−0.34***	−0.4***	−0.54***	1	0.34***	−0.34***
Days of poor physical health	−0.23**	−0.3***	−0.34***	0.27***	0.27***	0.34***	0.37***	0.54***	1	−0.55***	−0.2**	0.26***
Chronic health conditions	−0.02	−0.21**	−0.21**	0.09	0.11	0.19**	0.3***	0.29***	0.46***	−0.38***	−0.11	0.15*
Insomnia	−0.25***	−0.17*	−0.42***	0.31***	0.18*	0.15*	0.37***	0.25***	0.26***	−0.27***	−0.21**	0.28***
Days of poor mental health	−0.3***	−0.41***	−0.35***	0.42***	0.46***	0.54***	0.59***	1	0.54***	−0.4***	−0.29***	0.42***
Overall stress	−0.49***	−0.52***	−0.4***	0.52***	0.8***	1	0.48***	0.54***	0.34***	−0.43***	−0.48***	0.4***
Poor mental health	−0.41***	−0.48***	−0.55***	0.44***	0.47***	0.48***	1	0.59***	0.37***	−0.36***	−0.33***	0.36***
Physical activity	0.21**	0.16*	0.21**	−0.2**	−0.18*	−0.26***	−0.19**	−0.14*	−0.18*	0.24**	0.27***	−0.12
Tobacco use	−0.09	−0.17*	−0.22**	0.02	0.05	0.03	0.19**	0.1	0.03	0.06	0.1	0.17*
Risky drinking	−0.02	−0.08	−0.13	−0.01	−0.09	−0.13	0.13	0.01	−0.09	0.08	0.15	0.15*
Healthy diet	0.03	0.06	0.03	−0.1	−0.2**	−0.2**	−0.06	−0.05	0.02	−0.01	0.26***	−0.04
Sleep hours	−0.19**	−0.12	−0.14*	0.14*	0.18*	0.17*	0.22**	0.18*	0.13*	−0.23***	−0.14*	0.18**
Sleepy at work	−0.24***	−0.22**	−0.21**	0.22**	0.17*	0.14*	0.36***	0.21**	0.07	−0.13*	−0.2**	0.37***
Cognitive functioning limitations	−0.42***	−0.38***	−0.52***	0.45***	0.48***	0.46***	0.57***	0.43***	0.36***	−0.38***	−0.42***	0.45***
Work limitations	−0.3***	−0.37***	−0.4***	0.35***	0.35***	0.45***	0.4***	0.41***	0.38***	−0.39***	−0.39***	0.49***
Low productivity	−0.32***	−0.37***	−0.39***	0.4***	0.35***	0.38***	0.37***	0.41***	0.27***	−0.32***	−0.29***	1
Domain 5: Home, community, and society												
Life satisfaction	0.36***	1	0.33***	−0.24***	−0.38***	−0.52***	−0.47***	−0.41***	−0.3***	0.37***	0.37***	−0.37***
Financial insecurity	−0.36***	−0.34***	−0.4***	0.25***	0.37***	0.48***	0.43***	0.36***	0.17*	−0.32***	−0.35***	0.27***
Support outside of work	0.05	0.17*	0.2**	−0.02	−0.05	−0.06	−0.11	−0.01	−0.06	0.13	0.1	−0.08
Activities outside of work	0.13	−0.08	0.15*	−0.15*	−0.08	0.0	−0.13	−0.06	−0.12	0.15*	0.05	−0.19*

*P < 0.05, **P < 0.01, ***P < 0.001.

## DISCUSSION

In the context of the TWH program, it is widely recognized that one of the priority research areas and emerging issues is the need to acquire more and more data on workers’ well-being.^3,26^ Although this concept is extremely important in the context of occupational medicine/health and must be duly taken into account by prevention professionals to guarantee the best possible health and safety conditions in the workplace, for years, there has been much discussion about how to correctly interpret the well-being of workers, how to quantify it, and then use any measures of it within OSH management system to implement the effectiveness of prevention and protection measures and then make the work environment as “worker-friendly” as possible.^33^ Consequently, to achieve this ambitious goal, we must first understand what we really mean by workers’ well-being. Therefore, the first step can only be the definition of this concept that should be broad (i.e., that takes into account the multiple factors—occupational and nonoccupational—that might influence workers’ well-being), standardized (i.e., that allows the concept to be stated in an unambiguous, certain and comprehensible way) and recognized (i.e., that is considered as valid and effective by the scientific community). In our opinion, the definition provided by the NIOSH (to which we referred earlier), by meeting the abovementioned criteria, represents an essential starting point for addressing the issue of workers’ well-being and building revitalized prevention strategies and policies around it.^34^ These new tools are necessary to ensure that the OSH system evolves to keep pace with changes in the world of work,^12,35^ thus enabling it to contribute to the protection of workers (now and in the future) through the application of a holistic approach that focuses on the status of workers in terms of health, satisfaction, and flourishing.^36,37^

However, for well-being to be more than a conceptual objective, it should be made operative. In this regard, while the definition of the concept is certainly a necessary condition, it alone cannot be sufficient to make it operational. Thus, it is essential to have appropriate instruments to measure the well-being of workers. In this context, the recent validation of the WellBQ^26^ represents a considerable step forward since, although this tool still has several limitations (mainly due to its recent development, and thus still limited use),^27^ its extensive application in different workplaces and working populations promises to gather useful information to develop evidence-based actions and interventions that are able to improve workers’ well-being and, at the same time, help address the current limitations of the WellBQ.^6^

Overall, the data obtained from our study show that the Italian WellBQ is easy and straightforward to use and does not pose any particular interpretive difficulties for Italian workers. Indeed, the items of the original instrument (that are created by the NIOSH researchers or drawn/adapted from the public domain or adopted, with permission when necessary, from existing questionnaires) are all extremely clear, logical for the objectives of the questionnaire and reasoned in their placement within the different domains. In our opinion, these features, taken together, allow authors/translators with experience in occupational medicine to translate the original instrument quite easily. Not surprisingly, the comparison of the two forward translation versions did not reveal any particularly striking differences. In some questions, there were differences in the use of one synonym rather than another, but overall, the meaning of the items remained substantially unchanged and clear to the reader. Likewise, the two back translations returned English versions of the questionnaire that were very similar to the original WellBQ in terms of semantics and content consistency. Minor discrepancies were clarified through a consensus among the expert committee. Therefore, all in all, the work carried out by the expert committee was not particularly difficult as it consisted mainly in settling the choice between similar terms that were content-wise more in keeping with the original instrument but, at the same time, also more easily comprehensible and culturally akin to the Italian context. From this perspective, to obtain a translation of the WellBQ that is faithful to the original and effective in measuring workers’ well-being, it is essential that the translation be preceded by careful reading of the entire document presenting the questionnaire and its various paragraphs (e.g., development of the instrument, content of the domains, instructions for administration, coding system and scoring) to get an overall view of the questionnaire and valuable information that can help the translator keep the meaning of the various items intact.

With regard to the activities carried out by the expert committee, once the Italian final version of the tool was defined, particular attention was paid to verifying whether the items were actually applicable (and to what extent) in the Italian working context or whether they needed specific adaptations or precautions. Overall, after a careful evaluation of the questions, at the end of these reflections all the items of the questionnaire were judged relevant. Obviously, concerning some questions, the social, cultural or legislative context can be quite different from the American one but, in general, these differences can be reflected in the different response frequencies provided by the participants but not in the applicability of the questions. For example, concerning the question of the benefits offered by employers, and in greater detail with regard to health insurance, it should be pointed out that this possibility can actually be made available by some employers but usually these are large companies, while in Italy most of the companies are small and medium-sized. Therefore, in other words, the question is completely legitimate and applicable but the frequency of answers can be strongly influenced by the specific work context.

In this regard, it should be noted that this is a monocentric validation study that recruited similar workers, all belonging to the health care sector. Then, it is not possible to exclude that there might be differences in the interpretation of the questionnaire if it was administered to workers from different geographical areas (urban vs rural) or with different educational levels (high school vs bachelor’s or higher degree). However, it is equally important to underline that, as mentioned earlier, the items proposed by the WellBQ are very clear, direct, explicit, and examine different aspects and dimensions of well-being with which Italian workers are usually comfortable and familiar. Therefore, although differences in interpretation of the questionnaire cannot be ruled out, we assume that demographic, social or cultural differences may have an influence mainly on how the different items are answered rather than on their interpretation.

The validation model used demonstrates that the Italian version of the questionnaire is faithful to the original instrument and, as such, allows for efficient and robust measurement of worker well-being. In greater detail, CFI and TLI values ranged from 0.96 to 0.99, indicating excellent fit. Similarly, RMSEA values demonstrated a very close fit being all ≤0.06 for domain 1, 2 and 4, and 0.07 for domain 3. However, as detailed earlier in the results section, it should be kept in mind that some subdomains, constructs and single items were not included in the validation model. In this regard, we have already explained that the reasons for the aforementioned exclusions are fundamentally statistical in nature and mainly due to the detection (after applying the coding system) of low variability in the frequency of response to some specific questions such as those concerning injuries, alcohol consumption, work-related sexual harassment or physical violence. With respect to internal consistency of scales, measured by Cronbach’s α, again the data obtained are more than satisfactory since most of them showed values considerably higher than 0.7. Nevertheless, in some cases, such as for the “meaningful work”, “job engagement”, “physical activity”, and “financial insecurity” scales these values were suboptimal (e.g., 0.66, 0.53, 0.53, and 0.62, respectively) thus suggesting a moderate correlation between the individual items of these scales that, we have previously speculated, may be attributable to the specific inherent characteristics of the studied working population and the related workplace setting. Moreover, it should also be considered that all of these scales (with the exception of the “meaningful work”, which is a three-item scale) consist of only two items. In any case, these inconsistencies would not seem particularly troubling and, all in all, considering the results in their complexity, would not seem to impair the overall validation of the Italian version of the WellBQ.

In addition, following the example provided by Chari et al,^26^ to confirm the concurrent, convergent, and discriminant validity of the Italian version of the WellBQ, we also investigated the correlations among select scales and items of the questionnaire. The coefficients for these correlations observed in our study (Table 1) largely overlap with those provided in the aforementioned study and therefore further corroborate the validity of the Italian instrument. For example, with regard to concurrent validity, if we take the “job satisfaction” parameter as a reference, the correlations of this single item with the other items, indices, and scales of domains 1 to 3 go in the direction one would have expected basing on the information and data already published in the literature.^38^ In other words, “job satisfaction” is positively (and in the vast majority of cases even significantly) correlated with the other parameters that have a recognized positive influence on working conditions (e.g., wage, benefits and advancement satisfaction, supervisor and coworker support, supportive work culture, management trust, overall workplace safety, workplace safety climate), and conversely, it has negative and significant correlations with the elements that can negatively affect workers’ job satisfaction (e.g., work-related negative affect and fatigue, work to nonwork conflict, discrimination and work-related bullying). Moreover, from a validity perspective, it is interesting to note that the pattern of correlations of the “work-related positive affect” scale is completely identical to that just described for “job satisfaction”, while on the other hand, as it was logical to assume, the “work-related negative affect” scale exhibits an absolutely mirror-image pattern with respect to “work-related positive affect.”

A further confirmation of concurrent and discriminant validity of the questionnaire comes from analysis of the “life satisfaction” single item and comparison of the magnitude of its correlations with those of the “job satisfaction” parameter. Indeed, as would be expected, “life satisfaction” had positive correlation with the degree of “support outside of work”, and it is negatively associated with the “financial insecurity” scale, although surprisingly and differently from the study of Chari et al,^26^ we found a negative correlation between “life satisfaction” and “activities outside of work” (but it should be considered that this is a very weak and not statistically significant association, thus suggesting that its meaning is negligible). In addition, analysis of correlations between “life satisfaction” and key “health status” indicators reveals a largely assumed pattern showing, for example, positive associations with “overall health” and “physical activity” and negative ones with “days of poor physical and mental health”, “chronic health conditions”, “overall stress”, and “cognitive functioning or work limitations.” Evidence of discriminant validity comes from comparative analysis of the magnitude of correlations of “job satisfaction” and “life satisfaction” parameters with the other items in the questionnaire. In fact, generally speaking, associations of “job satisfaction” with domains 1 to 3 parameters related to working conditions are stronger than the correlations of life satisfaction with these elements, whereas conversely associations of “life satisfaction” with domain 5 measures are stronger or in the same order of magnitude (i.e., financial insecurity) than the associations of “job satisfaction” with the same parameters.

Finally, also with regard to convergent validity, our results are comparable and very similar to those obtained in the validation study of the original WellBQ.^26^ Indeed, the associations between different items/scales detecting several aspects of negative mental health (for example “work-related negative affect”, “overall stress”, “poor mental health”, and “poor mental health days”) are strong (all coefficients for correlations are ≥0.42) but at the same time they are far enough away from unity thus ensuring that they are conceptually different. Similar findings were observed also for correlations between “overall health” and “days of poor physical and mental health” or “chronic health conditions.”

## CONCLUSIONS

Overall, the results showed that the version of the WellBQ validated in this study possesses more than satisfactory psychometric qualities thus suggesting its reliability for use in Italian working populations and workplaces to measure and assess the workers’ well-being. In this regard, apart from a few exceptions that we have discussed extensively above, our statistical analyses revealed a good model fit and internal consistency. Furthermore, the evaluation of different correlations between several parameters of the questionnaire supported the concurrent, convergent, and discriminant validity of this instrument by providing data fully consistent with the main theories and expectations of occupational medicine and health. In this context, it is worth pointing out that our results are very similar and practically superimposable to those obtained by Chari et al,^26^ and therefore, reinforcing what has already been described by the creators of WellBQ and representing an indirect confirmation of the reliability and methodological rigor of the original WellBQ, they can only strengthen its extensive application. In this perspective, findings provided by this study represent an important contribution that can be useful in improving current knowledge on the topic of workers’ well-being.

In particular, the validation of WellBQ in Italian language will allow it to be widely used by Italian native speakers and in this respect, considering that the guidance provided by the Italian PNP 2020–2025 encourages and exhorts the implementation of preventive strategies based on the TWH model, our data represent an unavoidable starting point to arrive, with time and further studies, at a valid, informed, and effective use of the WellBQ in Italy to inform shared decision making and elicit areas where workers need more support.
